# Cardiac Computed Tomography as a Diagnostic Modality for the Assessment of Complex Congenital Heart Disease Management

**DOI:** 10.7759/cureus.16838

**Published:** 2021-08-02

**Authors:** Anshuman Darbari, Garima Sharma, Prashant L Patil, Barun Kumar, Rahul Sharma

**Affiliations:** 1 Department of Cardiothoracic & Vascular Surgery (CTVS), All India Institute of Medical Sciences, Rishikesh, IND; 2 Department of Radiodiagnosis, All India Institute of Medical Sciences, Rishikesh, IND; 3 Department of Cardiology, All India Institute of Medical Sciences, Rishikesh, IND

**Keywords:** atrial septal defect, partial anomalous pulmonary venous return, sinus venosus atrial septal defect, cardiac computed tomography, left superior vena cava

## Abstract

Here, we present a case of partial anomalous pulmonary venous return with the superior type of sinus venosus atrial septal defect. This case also had unusually persistent left-sided superior vena cava, which could not be diagnosed well in preoperative transthoracic echocardiography and required contrast-enhanced cardiac computed tomography scanning for proper diagnosing, operative planning, and avoidance of intraoperative problems. Postoperative, cardiac computed tomography scanning was also done to confirm adequate management.

## Introduction

Partial anomalous pulmonary venous return (PAPVR) is commonly associated with a superior type of sinus venosus atrial septal defect (SV-ASD). The incidence of PAPVR is approximately 0.5% and is more commonly seen in the right lung than the left lung. The anomalous pulmonary vein/s may drain into the right superior vena cava, azygous vein, inferior vena cava, coronary sinus, or right atrium. PAPVR is can also be an incidental finding in an adult with volume overload to the right-sided heart chambers due to left to right shunting of blood. Although persistent left superior vena cava (LSVC) is a rare anomaly, still, it is the most common and asymptomatic anomaly of thoracic veins. LSVC is frequently associated with other congenital anomalies, viz. PAPVR, atrial septal defect (ASD), atrioventricular septal defect (AVSD), and tetralogy of Fallot (TOF) [1-2].

Right from the understanding of congenital heart diseases, diagnostic techniques have improved over the years. Transthoracic echocardiography (TTE) and advanced transesophageal echocardiography (TEE) techniques have inherent limitations. Due to the poor acoustic window, both modalities may not be sensitive to diagnose all complex congenital structural abnormalities defects and associated anomalies. Especially for LSVC, it requires a high index of suspicion if a dilated coronary sinus is evident. Although TTE is the most commonly used diagnostic tool due to its easy availability, non-invasive profile, and fair accuracy, Contrast-enhanced cardiac computed tomography (CECCT) scanning is also important and invaluable in diagnosing and visualizing various complex congenital heart diseases [3].

## Case presentation

**Figure 1:**
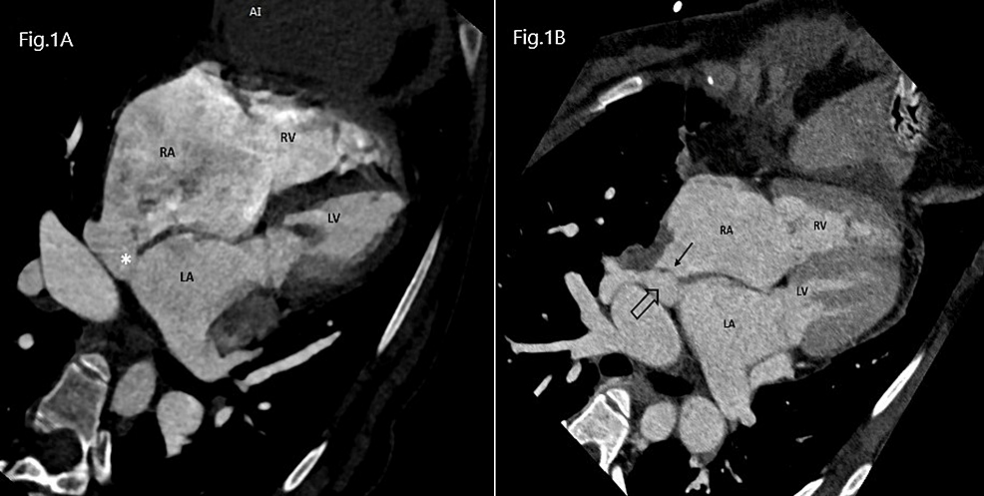
Section A: Contrast-enhanced CCT in axial four-chamber view showing a sinus venosus type defect in the interatrial septum (asterisk). Section B. Contrast-enhanced CCT in axial four-chamber view showing patch closure of atrial septal defect (thin black arrow) with pulmonary vein opening in the left atrium (thick black arrow) CCT: Cardiac Computed Tomography, RA: Right Atrium, LA: Left Atrium, RV: Right Ventricle, LV: Left Ventricle.

**Figure 2:**
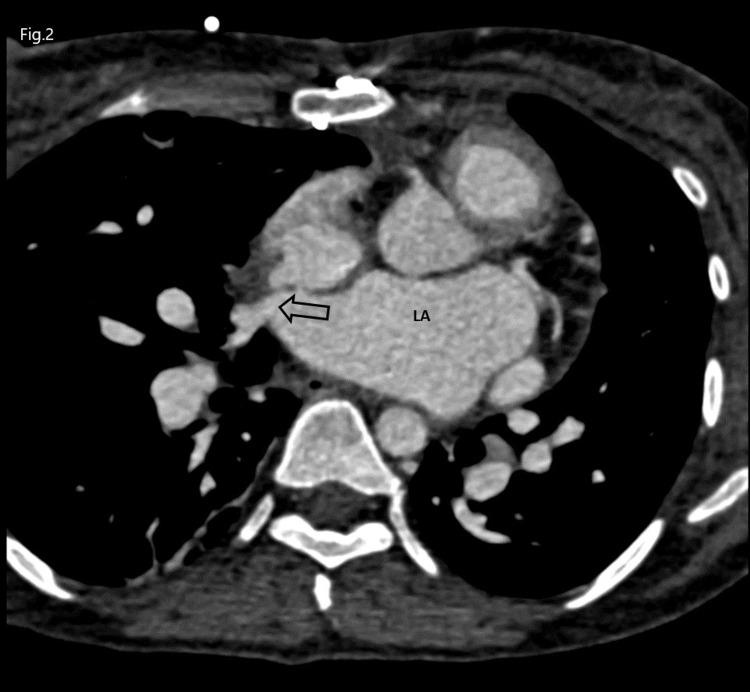
Contrast-enhanced CCT in axial four-chamber view showing right pulmonary vein opening in the left atrium after rerouting (thick black arrow) CCT: Cardiac Computed Tomography, LA: Left Atrium

**Figure 3:**
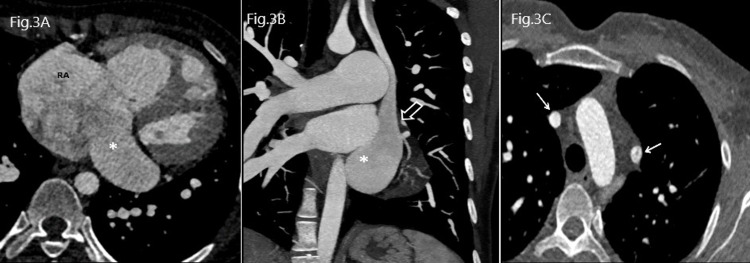
Section A: Contrast-enhanced CCT in axial view showing dilated coronary sinus (asterisk), Section B: Coronal maximum intensity projection (MIP) images view with dilated coronary sinus (asterisk) and left-sided superior vena cava draining into it (open white arrow), Section C: Contrast-enhanced CCT in axial view showing duplicated superior vena cava (white arrows) CCT: Cardiac Computed Tomography

**Figure 4:**
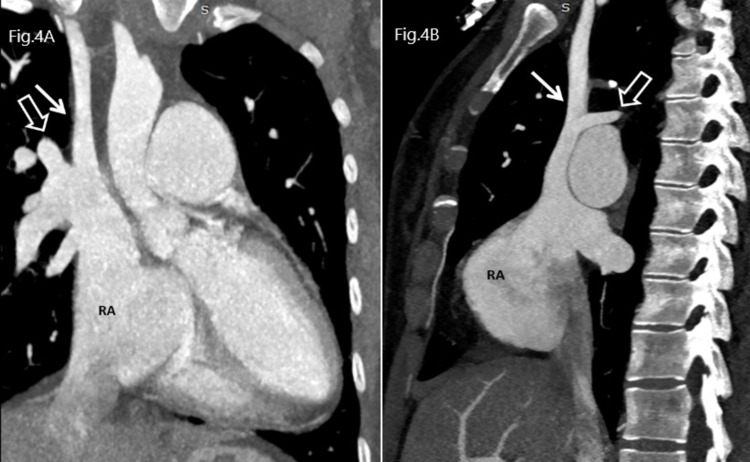
Sections A and B: Contrast-enhanced coronal and sagittal maximum intensity projection (MIP) images show the anomalous return of the right upper pulmonary veins (open white arrow) in the right superior vena cava (white arrow) RA: Right Atrium

A 33-year-old female patient presented with progressive dyspnea of New York Heart Association (NYHA) functional classification I and occasional palpitations associated with exertion for the last three months. The previous history was nonconclusive. On cardiac examination, the patient had loud P2, grade III ejection systolic murmur, and wide fixed split S2. Electrocardiography showed right axis deviation with incomplete right bundle branch block. Primary screening TTE suggested PAPVR with a secundum type of ASD. For detailed diagnosis and operative plan, TTE and TEE were done by a specialist. It was reported now as right-sided PAPVR with a superior type of SV-ASD of approximately 34 mm size with a dilated coronary sinus of 18 mm size. To get a better picture, we decided to get a CECCT scan. This study clarified the whole pathological anatomy and demonstrated right upper pulmonary veins draining into right-sided SVC, causing PAPVR with a superior type of SV-ASD of 35 mm with anomalous persistent LSVC draining to the right atrium via a dilated coronary sinus of 22 mm diameter (Figures 1-4).

The hematological studies were within normal limits. Under general anesthesia, by midline sternotomy, the operative procedure was started with standard aortic, right superior vena cava (RSVC), and inferior vena cava cannulation and snugging around the cannula. After standard cardioplegic arrest, the incision into the right atriotomy was extended into the RSVC. We found three separate pulmonary veins (PVs) were directly draining to the RSVC with superior type SV-ASD of 35 mm. Cardiotomy suction through the coronary sinus was used for LSVC drainage and to achieve a clear surgical field. SV-ASD closure was done with a glutaraldehyde-treated pericardial patch with rerouting and baffling of all pulmonary veins to the left atrium. After this, right-sided RSVC to right atrial junction augmentation was also done with a pericardial patch at the time of right atriotomy closure to avoid any stenosis. The postoperative phase was uneventful. Postoperative CECCT was done, which confirmed proper rerouting of PVs without any stenosis and adequate closure of SV-ASD and unobstructed, persistent LSVC with drainage to the right atrium. The patient was discharged on the eighth postoperative day in satisfactory condition.

## Discussion

The exact frequency of LSVC is not known because LSVC is often asymptomatic and detected incidentally. The prevalence of LSVC varies from 0.2% to 3% in the general population. There is no sex predilection for this anomaly. Generally, persistent LSVC is responsible for approximately 20% of the total venous blood return from the left arm, with the left side of the head and neck region. LSVC drains into the right atrial in 80%-90% of cases while the anomalous drainage towards the left atrial accounts for the remaining cases. Commonly, it drains into the right atrium through the coronary sinus opening without any hemodynamic significance. In these cases, the coronary sinus generally dilates and expands. As per available literature, there is a wide range of information about the association of cardiac anomalies with LSVC. The clinical significance of LSVC mainly depends on its cardiac drainage site and associated anomalies. The knowledge of LSVC is very fundamental in open-heart surgical procedures for bloodless, dry surgical fields and could be potential for an on-table surprise with a cardiopulmonary bypass plan change, especially in venous rerouting procedures, cavopulmonary anastomosis operations, and heart transplantation. Thus, it is imperative to recognize this preoperatively and reported it clearly, even when it is an incidental finding [4].

ASD is the most common congenital anomaly detected in the adult age. It may present with dyspnoea, arrhythmia, palpitations, or as an incidental diagnosis during a regular check-up. Only 5%-10% of ASD patients have SV-ASD type defects. A superior type of SV-ASD may have a combination of PAPVR anomalies. Also, most of the patient with PAPVR has an atrial septal defect of any kind. The total anomalous pulmonary venous return anomaly is presented as an emergency and usually diagnosed in newborns but patients with PAPVR can remain asymptomatic for a longer duration. Henceforth, the management of PAPVR is entirely different and individualized. PAPVR management is based on clinical presentation and the presence of ASD with a degree of the left-to-right shunt. Surgical repair is clearly indicated for a hemodynamically significant left-to-right shunt causing right-sided ventricular volume overload. Other indications for surgery include simultaneous surgical repair of other significant cardiac lesions or repeated pulmonary infections. Without surgical repair, PAPVR with ASD patients with a Qp/Qs ratio greater than two may ultimately develop shunt reversal and Eisenmenger syndrome physiology. After this, surgery is contraindicated in these cases because of the very high surgical risk and the low likelihood of reversal of pulmonary vasculature remodeling [5-6].

It's very challenging to diagnose these concomitant anomalies and necessitates the use of newer sophisticated techniques like CECCT or cardiac magnetic resonance imaging (CMRI) in addition to the easiest technique of echocardiography. Usually, CMRI can reliably detect and delineate the SV-ASD and PAPVR. CMRI has some other unique advantages over other cardiovascular imaging techniques, as it does not give radiation exposure and usually does not require any contrast medium. But, on the other hand, this method gives low spatial resolution, susceptibility for artifacts, increased pixel size, and comparatively longer examination times than CECCT. In addition, there are known contraindications to CMRI, including claustrophobia, pacemakers, and metal substances in the body. Also, some patients who do not cooperate for breath-hold sequences may potentially impair the image quality, thus making it difficult to achieve proper anatomical image formation and definition. Postoperative TTE is also problematic, as it does not provide a good window for proper images, especially when assessing complex repair or intracardiac patch baffling, rendering it difficult to rule out significant surgical technical complications or residual problems. Catheter-based angiography may visualize the repair and can also reconstruct vessels but is relatively invasive [7-8]. Compared to these diagnostic studies, a CECCT scan is helpful for postoperative evaluations to clearly demonstrate the anatomy by the reconstruction of 3-D volume rendering images, helping understand the repairs and anatomy with patency or occlusion of the connected pulmonary veins, septal defect closure, and LSVC. Data processing and the exact formation of multidimensional images in CECCT can be time-consuming, but reconstructed images are extremely invaluable in planning and assessing the surgery [9].

## Conclusions

Each cardiac diagnostic study can provide different and valuable information but in patients with complex congenital heart disease, the choice of investigation is crucial, and this provides the proper diagnosis, planning, and later evaluation of the completion of surgical procedure. Our case highlights how CECCT is a very useful diagnostic technique in the preoperative and postoperative evaluation of our patient with this rare type of PAPVR and SV-ASD with LSVC.
